# Data on energy and economic evaluation and microbial assessment of anaerobic co-digestion of fruit rind of *Telfairia occidentalis* (Fluted pumpkin) and poultry manure

**DOI:** 10.1016/j.dib.2018.09.065

**Published:** 2018-09-27

**Authors:** S.O. Dahunsi, A. Olayanju, J.O. Izebere, A.P. Oluyori

**Affiliations:** a Faculty of Environment and Labour Safety, Ton Duc Thang University, Ho Chi Minh City, Vietnam; b Biomass and Bioenergy Group, Environment and Technology Research Cluster, Landmark University, Omu-Aran, Kwara State, Nigeria; c Department of Agricultural and Biosystems Engineering, Landmark University, Omu-Aran, Kwara State, Nigeria; d Physical Sciences Department, Landmark University, Omu-Aran, Kwara State, Nigeria

**Keywords:** Biogas, Biomass, Economics, Energy, Fluted pumpkin, Microorganisms

## Abstract

The data described in this article was obtained in an experiment designed for the generation of biogas from the anaerobic co-digestion of *Telfairia occidentalis* (Fluted pumpkin) fruit rind and poultry manure both of which currently constitute an environmental nuisance in the localities where they are found. The data presented in this article is on the use of combined heat and power (CHP) system to assess the energy and economic feasibility of applying thermo-alkali pretreatment procedures to one of the substrates (Fluted pumpkin) prior to anaerobic digestion. Also, the microbial characterization and succession pattern of important microbes during the anaerobic digestion process was evaluated and the data reported in this paper.

**Specifications table**

**Table:** 

Subject area	Microbiology and Biotechnology
More specific subject area	Environmental Biotechnology
Type of data	Tables
How data was acquired	Combined Heat and Power (CHP) System, Analytical Profile Index (API) kits (BioMerieux, Leon, France)
Data format	Analysed
Experimental factors	Produced thermal energy, produced electrical energy, thermal energy gain, thermal energy requirement, net thermal energy, electrical energy gain, electrical energy requirement, net electrical energy
Experimental features	Energy and Economic evaluation of anaerobic co-digestion of pretreated and non-pretreated fruit rind of *Telfairia occidentalis* (Fluted Pumpkin) and Poultry Manure
Data source location	Omu-Aran, Kwara State
Data accessibility	The data is available within the article body

**Values of the data**

• The data presented in this article reveals the energy and economic evaluation of the anaerobic co-digestion of fruit rind of *Telfairia occidentalis* (Fluted Pumpkin) and Poultry manure for biogas generation
• The data will serve as a precursor for further research on the economic assessment of biomass pretreatment prior to anaerobic digestion processes
• The data give further exposure on the necessity and feasibility of pretreatment of biomass prior to anaerobic digestion.
• More robust heat and power systems can be used to further explore the generated data from this study in order to apply the processes in industrial scale experiments.

## 1 Data

The combined heat and power (CHP) system was used to assess the energy balance and the economic feasibility of applying thermal and alkaline pre-treatment to *T. occidentalis* fruit rind using a 50 and 30% thermal and electrical efficiencies respectively (Table 1). Therefore, to determine the thermal energy requirement (TER) for thermal and alkaline pre-treatments of *T. occidentalis* fruit rind, the energy needed to raise the temperature of 35 g TS L^−1^ *T. occidentalis* fruit rind mixture from 25 to 55 °C was determined using the specific heat of water i.e. 4.18 kJ kg^−1^ °C^−1^ in order to evaluate the specific heat of the mixture while neglecting heat loss [1], [2], [3].

**Table 1:** Energy and economic evaluation for the anaerobic co-digestion of *Telfairia occidentalis* fruit rind and poultry manure.

**Energy parameters**	**Experiment A**	**Experiment B**	**Experiment C**
Produced electrical and thermal energy from combined heat and power (CHP)	1785 ± 0.01	1699 ± 0.02	1155 ± 0.02
Produced thermal energy (kWh t^−1^ TS)	1645 ± 0.02	1547 ± 0.01	498 ± 0.01
Produced electrical energy (kWh t^−1^ TS)	770 ± 0.01	563 ± 0.02	340 ± 0.02
***Thermal balance***			
*Thermal energy gain (kWh t^−1^ TS)	1147 ± 0.01	1049 ± 0.03	–
Thermal energy requirement (kWh t^−1^ TS)	1088 ± 0.02	1109 ± 0.03	–
Thermal energy requirement with 80% of heat recovery (kWh t^−1^ TS)	218 ± 0.02	210 ± 0.01	–
^#^Net thermal energy (kWh t^−1^ TS)	59 ± 0.02	−60 ± 0.02	–
Net thermal energy with 80% of heat recovery (kWh t^−1^ TS)	−929 ± 0.02	−839 ± 0.03	–
***Electrical balance***			
^$^Electrical energy gain	430 ± 0.01	223 ± 0.02	–
Energy for mixing during pretreatment	–	–	–
Net electrical energy	430 ± 0.01	223 ± 0.01	
***Economic evaluation***			
Cost of NaOH (є t^−1^ TS)			

**Remark**:*=difference of thermal energies produced by the pretreated experiment minus the untreated; ^#^=difference between the thermal energy gain and the thermal energy requirement for the thermo-alkaline pretreatment; a ^$^=difference of electricity energies produced by pretreated experiment minus the untreated.

To assess the electrical energy, only the electric energy used for the substrate mixing was considered neglecting the energy used during mechanical treatment since this was also done for the experiment without thermal and alkaline pre-treatment [4]. Table 2 shows the heat balance of different biomass previously anaerobically digested with thermal and alkaline pre-treatments procedures [5], [6], [7], [8], [9].

**Table 2:** Energy balances of thermal and thermo-chemical pretreatment procedures as applied to different substrates.

**Substrate**	**Condition of pretreatment**	**Increase in methane yield (m^3^ t**^**−1**^**TS)/operation mode**	**Biogas conversion**	**Surplus thermal energy (kWh t**^**−1**^**TS)**	**Thermal pretreatment requirements (kWh t**^**−1**^**TS)**	**Net heat energy (kWh t**^**−1**^**TS)**	**References**
*Telfairia occidentalis* fruit rind	Thermo-alkaline (55 °C; 4% NaOH (w/w); 24 h) Solid load: 35 g TS L^−1^	40/Batch mode	CHP: 35% electricity; 50% heat	1147	1088	59	Current study
	Thermo-alkaline (55 °C; 4% KOH (w/w); 24 h) Solid load: 35 g TS L^−1^	35/Batch mode	CHP: 35% electricity; 50% heat	1049	1109	−60	Current study
*Tithonia diversifolia* shoot	Thermo-alkaline (55 °C; 4% NaOH (w/w); 24 h) Solid load: 35 g TS L^−1^	53/Batch mode	CHP: 35% electricity; 50% heat	1176	1068	108	[10]
	Thermo-alkaline (55 °C; 4% KOH (w/w); 24 h) Solid load: 35 g TS L^−1^	30/Batch mode	CHP: 35% electricity; 50% heat	862	1150	−288	[10]
Peanut hull	Thermo-alkaline (55 °C; 4% NaOH (w/w); 24 h) Solid load: 35 g TS L^−1^	70/Batch mode	CHP: 35% electricity; 50% heat	761	1173	−412	[11]
Sunflower stalks	Thermo-alkaline (55 °C; 4% NaOH (w/w); 24 h) Solid load: 35 g TS L^−1^	36/Continuous mode	CHP: 35% electricity; 50% heat	185	1034	−849	[12]
	Thermo-alkaline (55 °C; 4% NaOH (w/w); 24 h) Solid load: 50 g TS L^−1^	36/Continuous mode	CHP: 35% electricity; 50% heat	185	733	−548	[12]
	hermo-alkaline (55 °C; 4% NaOH (w/w TS); 24 h) Solid load: 200 g TS L^−1^	36/Continuous mode	CHP: 35% electricity; 50% heat	185	210	−25	[12]
	Thermo-alkaline (55 °C; 4% NaOH (w/w); 24 h) Solid load: 50 g TS L^−1^ 80% of heat recovery from pretreatment	36/Continuous mode	CHP: 35% electricity; 50% heat	185	147	38	[12]
Sunflower Oil Cake	Thermal (170 °C; 1 h)	32/Batch mode	CHP: 35% electricity; 50% heat	161	3535	−3375	[6]
	Solid load: 50 g TS L^−1^						
	Thermal (170 °C; 1 h)	32/Batch mode	CHP: 35% electricity; 50% heat	161	1010	−849	[6]
	Solid load: 200 g TS L^−1^						
	Thermal (170 °C; 1 h) Solid load: 200 g TS L^−1^ 80% of heat recovery from pretreatment	32/Batch mode	CHP: 35% electricity; 50% heat	161	152	9	[6]
Ensiled Sorghum Forage	Thermo-alkaline (100 °C; 30 min, 10% NaOH w/w) Solid load: 160 g TS L^−1^	92/Batch mode	CHP: 40% electricity; 41% heat	378	547	−169	[13]
	Thermo-alkaline (100 °C; 30 min, 10% NaOH w/w) Solid load: 160 g	92/Batch mode	CHP: 40% electricity; 41% heat	378	109	269	[13]
	TS L^−1^ 80% of heat recovery from Pretreatment						
Wheat straw	Thermo-alkaline (100 °C; 30 min, 10% NaOH w/w) Solid load: 160 g TS L^−1^	137/Batch mode	CHP: 40% electricity; 41% heat	577	547	30	[13]
	Thermo-alkaline (100 °C; 30 min, 10% NaOH w/w) Solid load: 160 g TS L^−1^ 80% of heat recovery from Pretreatment	137/Batch mode	CHP: 40% electricity; 41% heat	577	109	468	[13]
Microalgae	Thermal (75 °C; 15 min) Solid load: 11.7 g TS L^−1^ 85% of heat recovery from Pretreatment	32/Batch mode	100% heat conversion	316	458	−142	[7]
	Thermal (75 °C; 15 min) Solid load: 20 g TS L^−1^ 85% of heat recovery from Pretreatment	32/Batch mode	100% heat conversion	316	268	48	[7]
	Thermal (75 °C; 15 min) Solid load: 30 g TS L^−1^ 85% of heat recovery from Pretreatment	32/Batch mode	100% heat conversion	316	173	143	[7]

In the co-digestion of *Telfairia occidentalis* fruit rind and poultry manure, various aerobic and anaerobes bacteria, fungi and methanogens were isolated and characterized (Table 3).

**Table 3:** Microbial evaluation and succession in the anaerobic co-digestion of *Telfairia occidentalis* fruit rind+poultry manure.

**Day**	**Aerobes (Cfu/ml)**		**Fungi (Cfu/ml)**		**Anaerobes (Cfu/ml)**		**Methanogens (Cfu/ml)**	
	**Organism**	**TAPC**	**Organism**	**TFC**	**Organism**	**TPC**	**Organism**	**TPC**
0	*Bacillus* sp.	2.3 × 10^10^	*Aspergillus niger*	1.0 × 10^8^	*Fusobacterium* sp.	1.2 × 10^10^	*Methanosarcinales* sp.	1.2 × 10^10^
	*Serratia* sp.		*Aspergillus flavus*		*Bacteroides* sp.		*Methanobacteriales* sp.	
	*Pseudomonas aeruginosa*		*Rhizopus* sp.		*Clostridium* sp.		*Methanomicrobiales* sp.	
	*Proteus* sp.		*Mucor* sp.		*Porphyromonas* sp.		*Aminobacteria* sp.	
			*Penicillum* sp.					
6 6	*Bacillus* sp.	1.4 × 10^8^	*Aspergillus niger*	1.2 × 10^8^	*Fusobacterium* sp.	1.0 × 10^6^	*Methanosarcinales* sp.	1.0 × 10^8^
	*Serratia* sp.		*Aspergillus flavus*		*Bacteroides* sp.		*Methanobacteriales* sp.	
	*Pseudomonas aeruginosa*		*Rhizopus* sp.		*Clostridium* sp.		*Methanomicrobiales* sp.	
	*Proteus* sp.		*Mucor* sp.		*Porphyromonas* sp.		*Aminobacteria* sp.	
			*Penicillum* sp.					
12 12	Nil	Nil	*Aspergillus niger*	1.0 × 10^3^	*Fusobacterium* sp.	1.0 × 10^4^	*Methanosarcinales* sp.	1.0 × 10^5^
			*Aspergillus flavus*		*Bacteroides* sp.		*Methanobacteriales* sp.	
			*Rhizopus* sp.		*Clostridium* sp.		*Methanomicrobiales* sp.	
			*Mucor* sp.		*Porphyromonas* sp.		*Aminobacteria* sp.	
			*Penicillum* sp.					
18 18	*Bacillus* sp.	1.0 × 10^2^	*Aspergillus niger*	1.0 × 10^2^	*Fusobacterium* sp.	1.3 × 10^10^	*Methanosarcinales* sp.	1.0 × 10^10^
					*Clostridium* sp.		*Methanobacteriales* sp.	
					*Porphyromonas* sp.		*Methanomicrobiales* sp.	
							*Aminobacteria* sp.	
24 24	*Bacillus* sp.	1.0 × 10^2^	*Aspergillus niger*	1.0 × 10^2^	*Fusobacterium* sp.	1.2 × 10^3^	*Methanosarcinales* sp.	1.7 × 10^10^
					*Clostridium* sp.		*Methanobacteriales* sp.	
					*Porphyromonas* sp.		*Methanomicrobiales* sp.	
							*Aminobacteria* sp.	
30 30	*Bacillus* sp.	1.0 × 10^2^	*Aspergillus niger*	1.0 × 10^2^	*Fusobacterium* sp.	1.2 × 10^2^	*Methanosarcinales* sp.	2.7 × 10^12^
					*Clostridium* sp.		*Methanobacteriales* sp.	
							*Methanomicrobiales* sp.	
							*Aminobacteria* sp.	

**Remark**: *TAPC=Total aerobic plate count; TFC=Total fungal count; TPC=Mean Plate Count.*

## 2 Experimental design, materials and methods

### 2.1 Materials and method

Data was obtained from the evaluation of pretreatment application to fruit rind of *Telfairia occidentalis* and the possibility of gaining back the investment (obtaining of chemicals and heat) into the pretreatment procedure through the sale of additional energy gained.

### 2.2 Experimental design

A simple computational equation was used to first determine the thermal energy required (TER) in kWh t^-1^ TS for raising the temperature of one ton TS of *T. occidentalis* fruit rind from 25 to 55 °C during pre-treatment [14], [15], [16].

### 2.3 Microbial enumeration

The aerobic organisms (Bacteria and fungi) associated with the fermenting substrates were isolated and enumerated weekly using standard methods [17], [18], [19]. Facultative anaerobes were serially isolated using specialized media in an anoxic condition at 37 °C for 5 to 7 days as earlier reported [20], [21]. Confirmation of the presumptive isolates was done with corresponding rapid Analytical Profile Index (API) kits [22] while a basal medium was used for identifying methanogens [23], [24].

### 2.4 Statistical data analysis

The paired sample t-tests were conducted to determine the significant difference in the means of three replicates.
