# Supplementary material for: Data on energy and economic evaluation and microbial assessment of anaerobic co-digestion of fruit rind of Telfairia occidentalis (Fluted pumpkin) and poultry manure
Source: Data Brief. 2018 Sep 27;21:97–104. doi: 10.1016/j.dib.2018.09.065 (PMC6186960; doi:10.1016/j.dib.2018.09.065)
Supplement: Supplementary file 1 — Supplementary material. [file mmc1.docx]

**Conflict of Interest**

Authors declare no conflict of interest whatsoever.
